# Interrelated dataset of rebound numbers, ultrasonic pulse velocities and compressive strengths of drilled concrete cores from an existing structure and new fabricated concrete cubes

**DOI:** 10.1016/j.dib.2023.109201

**Published:** 2023-05-05

**Authors:** Daniel Gebauer, Raúl Enrique Beltrán Gutiérrez, Steffen Marx, Marko Butler, Konrad Grahl, Thomas Thiel, Stefan Maack, Stefan Küttenbaum, Stephan Pirskawetz, Wolfgang Breit, Martin Schickert, Marco Krüger

**Affiliations:** a Technische Universität Dresden, Institute of Concrete Structures, Dresden 01062, Germany; b Technische Universität Dresden, Institute of Construction Materials, Dresden 01187, Germany; c Hochschule für Technik und Wirtschaft Dresden, Dresden 01069, Germany; d Bundesanstalt für Materialforschung und -prüfung (BAM), Berlin 12203, Germany; e Technische Universität Kaiserslautern, Construction Material Technology, Kaiserslautern 67663, Germany; f Materialforschungs- und -prüfanstalt an der Bauhaus-Universität Weimar, Weimar 99423, Germany

**Keywords:** Nondestructive evaluation, Existing structures, Rebound hammer, Ultrasonic pulse velocity, Compressive concrete strength, Interlaboratory test, Civil engineering

## Abstract

Two test series were examined using nondestructive measuring methods by six independent laboratories before determining their compressive strength. The nondestructive test methods used were the rebound hammer and ultrasonic pulse velocity measurement. Two types of geometries were investigated: drilled cores and cubes. The measurement procedure for each of these datasets is conditioned to the geometry and is therefore different.

The first series consists of 20 drilled cores (approximately diameter/height = 10 cm/20 cm) from the 55-year-old Lahntal Viaduct near Limburg, Germany. After preparation in the first laboratory, the lateral surface of the drilled cores was tested with the rebound hammer using a given pattern. Every laboratory tested every drilled core at different locations. Ultrasonic measurements in transmission were performed repeatedly at predefined points on the flat surfaces of the specimen.

The second series consisted of 25 newly manufactured concrete cubes of a mix with a target concrete strength class of C30/37. The edge length was 15 cm. Each laboratory received five specimens of this test series. Thus, contrary to the first series, each specimen was tested by only one laboratory. Two side faces of each cube were tested with the rebound hammer. In addition, ultrasonic measurements were performed by one laboratory. The time of flight was measured between the tested side faces of the rebound hammer at different positions.

For both series, rebound hammers were used to determine the R-value as well as the Q-value. The rebound hammer models within the laboratories were always the same, while they differed between the laboratories. The ultrasonic measurements took place with different measurement systems and couplants. Finally, both specimen series were tested destructively for compressive strength.

The dataset contains the raw data summarized in tabular form. In addition, relevant calculated data are included in some cases. For the ultrasonic measurements, the time of flight has already been converted into the ultrasonic velocity. Besides, in addition to the raw data of the compressive strength test (force, weight, and geometry values), the calculated compressive strengths and densities are also provided.

**Specifications Table**

**Table:** 

Subject	Civil and Structural Engineering
Specific subject area	Nondestructive evaluation of concrete (Rebound Hammer and Ultrasonic Measurement)
Type of data	Table (.csv)
How the data were acquired	Rebound Hammer The rebound numbers of clamped concrete specimens were measured using with N-type rebound hammers (impact energy: 2207 Nm). Overall, the SilverSchmidt (Q-values) and OriginalSchmidt (R-values) models were used. The raw data were directly read, stored, and summarized in tabular form. Ultrasonic Measurements Repeated transmission measurements of ultrasonic pulse velocity were conducted on both the core and cube specimens. Various transducers with respective central frequency were used (20–95 kHz). An additional homogeneous polyamide specimen was tested in the same way for verification.
Data format	Raw, partially processed by calculations
Description of data collection	1. Specimens were preserved in a temperature and humidity-controlled chamber (20 ± 2 °C and 65 ± 5% RH) before and after the experiments. 2. Positions of the measurements were precisely specified. 3. Result values to be provided were defined uniformly for all laboratories. 4. Data collection was carried out in accordance with German standards (DIN EN 12,504–2 [1], −4 [2]). Furthermore, each laboratory was given the freedom to use their own common procedure for determining the measurement values.
Data source location	*Institution:* Technische Universität Dresden, Institute of Concrete Structures *City:* Dresden *Country:* Germany
Data accessibility	Repository name: Harvard Dataverse Data identification number: 10.7910/DVN/AFCITK Direct URL to data: https://doi.org/10.7910/DVN/AFCITK[3]

## Value of the Data

• The availability of well-classified and systematic obtained results from nondestructive and destructive testing evaluation methods is essential for the efficient evaluation of NDE data and test results. Currently, civil engineers, contractors, laboratories, and government agencies do not have reference values to evaluate the accuracy of destructive and nondestructive test data sets from existing constructions. This dataset fills this gap.
• This dataset comprises measurement data from six independent laboratories, utilizing up to three measurement techniques on two types of concrete. It facilitates comparisons within and across laboratories, between concrete types, and measurement techniques. Precision values such as repeatability and reproducibility can be evaluated, including for structural and laboratory concrete using the rebound hammer, and for structural concrete using ultrasonic data.
• For each nondestructive value, the corresponding concrete compressive strength value from destructive testing is available, so that the correlation of destructive and nondestructive parameters can be evaluated. For structural concrete, the correlation between the nondestructive methods themselves is also possible. The data can be used to verify and improve existing and future correlation functions between nondestructive and destructive data. Especially with regard to old concrete, hardly any data sets are available.
• The beneficiaries of this data set are scientists and research institutions in the fields of concrete construction, building material testing, and non-destructive testing. They are provided with comprehensive, coherent data that can be processed according to their requirements. The test design can support and expedite future test designs and can also serve as an example. This applies both to comparative tests for the precision of scientific institutions and to simple material tests performed by companies.
• Reference values of the precision can be obtained from the data, contributing to reduce experimental errors during measurement campaigns involving different operators or laboratories. Currently, there are no such reference values, as can be seen in European standardization (DIN EN 12,504–2 [1] and DIN EN 12,504–4 [2]). Therefore, standardization bodies also benefit from the data obtained.
• By determining assured precision values, the quality of the measurement procedure can be assessed, and its suitability for certain measurement tasks can be evaluated. The precision values provide information about the scatter and uncertainty of the measurement method under consideration and can be taken into account in the context of the semi-probabilistic safety concept in civil engineering.

## 1 Objective

Currently, it is unclear what precision can be achieved with nondestructive testing methods when testing hardened concrete. Most published datasets are abbreviated, the boundary conditions are sometimes unclear, and often aim only at correlation between nondestructive test values and concrete compressive strength. This dataset was created to estimate the repeatability and reproducibility of the nondestructive test methods studied. The evaluation of specimens under pre-established and equal conditions for all laboratories provides a data basis for estimating the inter-laboratory reference accuracy for in-situ concrete specimens. Subsequently, the extensive dataset collected can also be used to investigate the correlation between nondestructive data and the strength of destructively tested concrete.

## 2 Data Description

The dataset contains only data summarized in tables. The files were created using Excel (Version 2108) and have the file format ".csv" (comma separated values), which can be read by most evaluation programs. The designation and structure of each file is described below.

### 2.1 General file designation

The files are named according to the following rule:

*Type of specimen_Measurement method(_Additional description)*

**Table:** 

Type of specimen:	“core” – Drilled cores from an existing structure
	“cube” – Newly fabricated cube specimens
Measurement method:	“rn_R” – Rebound number (R-Value)
	“rn_Q” – Rebound number (Q-Value)
	“us” – Ultrasonic data
	“compressive strength” – Compressive strength test data
Additional description:	“summary” – Summarized table for all laboratories
	“lab1” to “lab6” – Single laboratory content
	“designation key” – Key for the table designations

### 2.2 Rebound hammer data

The dataset of the rebound hammer contains the raw rebound number (rn), which can be either an R-Value or a Q-Value depending on the type of rebound hammer used. The values itself have no unit, but to differentiate between measurement principles, the letters “R” and “Q” are used. The data are summarized in two tables separated according to the rebound hammer type and provided in “.csv”-format. Each value can be clearly assigned to a specimen and a laboratory.

The core tables are organized as follows:

• Header (rows 1 – 4)
  ○ Row 1: Number of the executing laboratory (Lab 1 – Lab 5)
  ○ Row 2: Type of rebound number value (R or Q)
  ○ Row 3: Position of the tested column on the lateral surface in [°]
  ○ Row 4: Number of the tested line on the lateral surface in Roman numerals (I – VI)

• Describing Columns (columns 1 – 2)
  ○ Column 1: Number of the core
  ○ Column 2: Core name as original identifier

• Actual raw data of rebound numbers
  ○ Between rows 5 – 24 and columns 4 – 39

The cube tables are organized as follows:

• Header (rows 1 – 3)
  ○ Row 1: Type of rebound number value (R or Q)
  ○ Row 2: Position of the tested column on side face named in Latin letters (A – E)
  ○ Row 3: Number of the tested line on the side face in Roman numerals (I – V)

• Describing Columns (column 1 – 3)
  ○ Column 1: Number of the executing laboratory (Lab 1 – Lab 5)
  ○ Column 2: Number of the cube (W01 – W25)
  ○ Column 3: Tested side face (S1 – S2)

• Actual raw data of rebound numbers
  ○ Between rows 4 – 28 and columns 5 – 29

### 2.3 Ultrasonic data

The ultrasonic testing consisted of 12 successive measurements of the time of flight. Two types of measurement points were defined: Center Points (CP) and Third Points (TP). The data in the table have been sorted according to the following layout.

• Header (rows 1 – 4)
  ○ Row 1: Measuring point: Point type CP or TP. The number after CP represents the measurement repetition at that point, e.g., CP5 (fifth repetition). The number after TP represents the measured point position TP2
  ○ Row 2: Azimuth angle in [°] of the measurement point TP with respect to the reference meridian plane of the specimen
  ○ Row 3: Parameter: e.g., Laboratory, TOF: time of flight, UPV: Ultrasonic Pulse Velocity,
  ○ Row 4: Unit of the measured parameter

• Describing Columns
  ○ Column 1: Number of the core
  ○ Column 2: Core name as original identifier
  ○ Column 3: Laboratory
  ○ Column 4 – 12: Specimen data
  ○ Column 13 – 14: Measurement conditions
  ○ Column 15 – 38: Measurement data TOF-UPV

### 2.4 Compressive strength test

The dataset of the compressive strength values contains the raw data of the geometry, weight and force as well as the calculated values of the density and compressive strength. The machine force is divided by the measured cross-sectional area of the specimens. The dataset contains the geometric dimensions of each specimen as well as the calculated compressive strength.

The data are presented in tables and given in “.csv”-format. Each value can be clearly assigned to a specimen.

The core tables are organized as follows:

• Header (rows 1 – 2)
  ○ R. 1: Name of the values contained in the column
  ○ R. 2: Unit of the values contained in the column

• Columns
  ○ C. 1: Number of the core
  ○ C. 2: Core name as the original identifier
  ○ C. 3: Diameter as the mean out of three measured values
  ○ C. 4: Hight as mean out of three measured values
  ○ C. 5: Area of the core calculated from the diameter
  ○ C. 6: Weight of the specimen
  ○ C. 7: Density calculated from weight divided by the volume of area and height
  ○ C. 8: Maximum force at failure
  ○ C. 9: Compressive strength calculated from force divided by area

The cube tables are organized as follows.

• Header (rows 1 – 2)
  ○ R. 1: Name of the values contained in the column
  ○ R. 2: Unit of the values contained in the column

• Columns
  ○ C. 1: Number of the cube
  ○ C. 2: Length of the first side of the cube as the mean out of four values
  ○ C. 3: Length of the second side of the cube the as mean out of four values
  ○ C. 4: Length of the third side of the cube as the mean out of four values
  ○ C. 5: Area of the cube calculated from the first and second length
  ○ C. 6: Weight of the specimen
  ○ C. 7: Density calculated from the weight divided by the volume of area and height
  ○ C. 8: Maximum force at failure
  ○ C. 9: Compressive strength calculated from the force divided by area

## 3 Experimental Design, Materials and Methods

### 3.1 Specimens

#### 3.1.1 Drilled core series

**Fig. 1:**
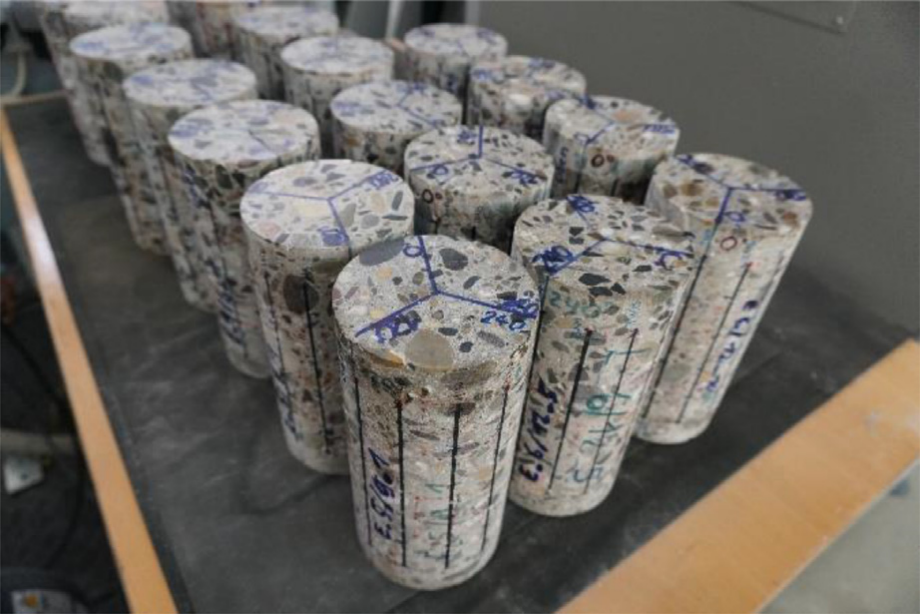
Drilled cores prepared for testing.

The first test series contains 20 drilled cores from 55-year-old bridge. Their diameter is 100mm, their height is 200mm, and the documented concrete class was B450. The cores were obtained by wet drilling and then the end planes were prepared by cutting and wet grinding. Until the beginning of the tests the specimens were stored in a climatic chamber under standard conditions (20 °C/65% rH). The prepared cores can be seen in Fig. 1. All samples fulfill the requirements according to DIN EN 12,504–1 [4].

#### 3.1.2 Cube series

**Fig. 2:**
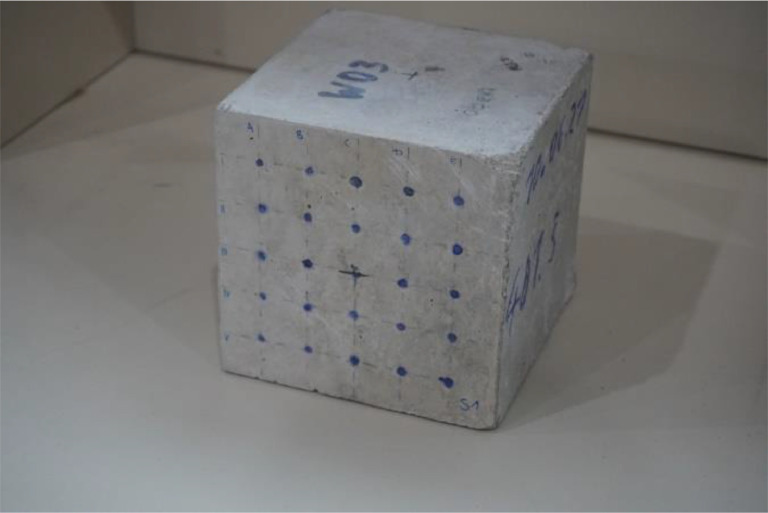
Cube prepared for testing.

The second test series consists of 25 cube specimens manufactured according to the standard DIN EN 12,390–3 [5] with a concrete class of C30/37. The edge length is 150mm. The prepared cube can be seen in Fig. 2.

## 4 Measurement Systems

### 4.1 Rebound hammers

Table 1 shows an overview of the measurement systems used for each laboratory. The rebound hammer “Silver Schmidt” returns the rebound value Q while all other return the rebound value R. All rebound hammers are “Typ N”, which means that they have a impact energy of 2207 Nm.

**Table 1:** Measurement systems for rebound number acquisition.

	Method	Modell
Lab 1	R, Q	Proceq DIGI-SCHMIDT 2000 (Modell ND), Proceq Silver Schmidt OS8200 N (Typ N)
Lab 2	R	Proceq Original Schmidt (Typ N)
Lab 3	Q	Proceq Silver Schmidt (Typ N)
Lab 4	R, Q	Proceq Silver Schmidt (Typ N), Proceq Original Schmidt (Typ N)
Lab 5	R, Q	Proceq Original Schmidt (Typ N), Proceq Silver Schmidt (Typ N)
Lab 6	–	–

### 4.2 Ultrasonic measurement

Table 2 shows an overview of the ultrasonic measurement devices and transducers used by each laboratory. Before the measurement campaign, each laboratory used the same standard calibration bar to offset the ultrasonic measurement equipment to a reference value.

**Table 2:** Measurement systems for ultrasonic data acquisition.

	Ultrasonic Equipment	Software	Transducer	Center frequency	Couplant
Lab 1	Ritec RAM 5000	RAM-5000 v 1.1.3	Olympus V101-RB	95 kHz	Acrylpads
Lab 2	Geotron USG 40	Lighthouse *	UP-80	80 kHz	Acrylpads
Lab 3	Geotron Lighthouse	Lighthouse *	UPG-D/UPE-D	46 kHz	no couplant
Lab 4	Geotron USG 40	Lighthouse*	UPG-D/UPE-D	64 kHz	no couplant
Lab 5	Proceq pundit	Pundit Lab (+)	Pundit Transducer 54 kHz	54 kHz	Acrylpads
Lab 6	Geotron USG 20, Fluke 192B	Lighthouse *	UPG-D/UPE-D	20 kHz	no couplant

⁎ The Lighthouse software is customized for each device.

## 5 Data Acquisition

### 5.1 Rebound numbers on cores

**Fig. 3:**
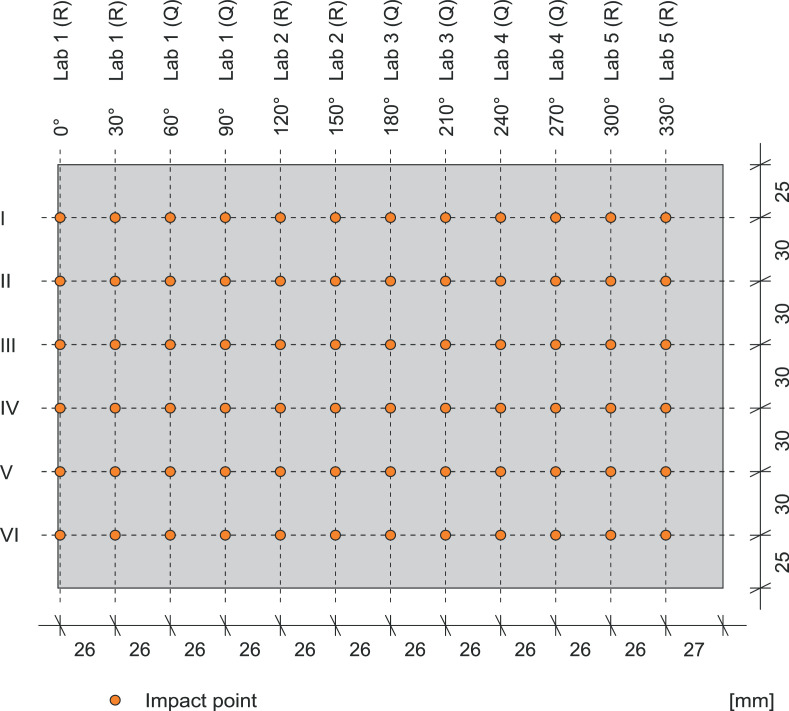
Unrolled lateral surface of a drilled core with impact pattern.

The area of testing is the lateral surface of the drilled core. This area is divides by each 30°, which leads to 12 vertical lines. On every line, 6 impact points were marked with a distance of 30mm and numbered with the Roman numerals from I to VI. The outer impact points have a distance of 25mm from the edge. Fig. 3 shows the unrolled lateral surface of a core with the corresponding impact pattern. Each point may only be hit once. Each laboratory tests each core, resultingin a dataset of 12 rebound numbers per laboratory and cube and 60 rebound numbers in total per core. During the data acquisition, the drill cores were fixed in a testing machine with 100kN to secure their position. All rebound hammers were checked for calibration on the test anvil before and after the test and passed den test.

### 5.2 Ultrasonic pulse velocity on cores

The measurements were performed under laboratory conditions at a temperature of 20 °C (±2 °C) and a relative humidity of 65% (±5%). Low-frequency transducers (center frequency from 20 to 95kHz) were used for the investigation. Before the measurement campaign, each laboratory used the same standard calibration bar to offset the ultrasonic measurement equipment to a reference value. For this reference measurement, the same coupling and the same measuring conditions were used as for the subsequent measurement on the concrete specimens. Each laboratory used a special apparatus to ensure a constant controlled contact pressure between the specimen and the transducers. The measurements were carried out in compliance with the recommendations for transmission measurements prescribed in DIN EN 12,504–4 [2] and in the technical bulletin B04 2018 of the German Society for Nondestructive Testing [6].

**Fig. 4:**
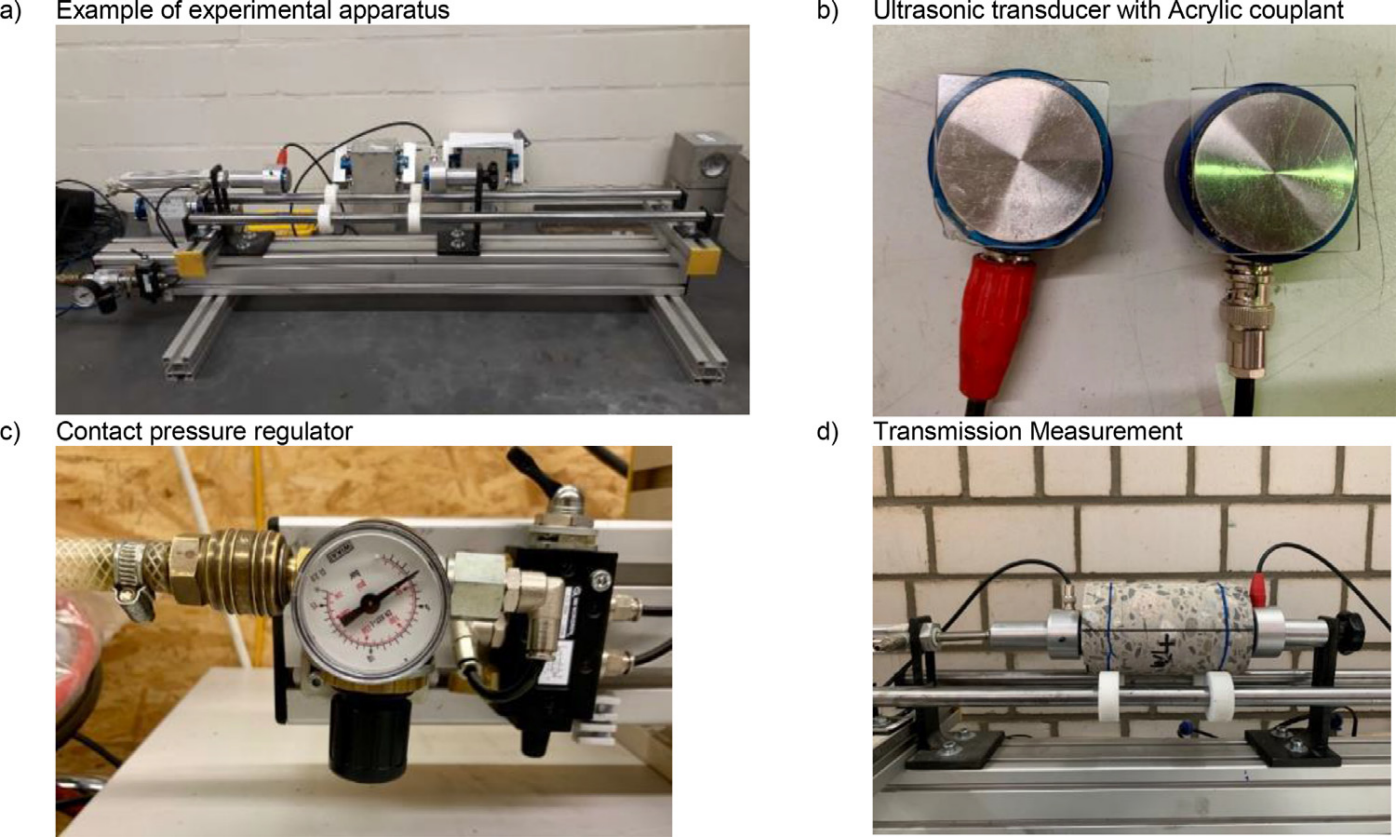
Exemplary preparations for measurement at laboratory 1.

The ultrasonic measurements, including the calibration, were carried out using a test apparatus in which the specimen can be firmly held to ensure a constant contact pressure of approximately 1bar ±0.5bar between transducer and specimen. All laboratories except Lab 4 and Lab 6 used Acryl pads as a couplant. Lab 4 and Lab 6 used dry coupling for the investigation. The ultrasonic transducers were applied to the front surface with a constant contact pressure (see Fig. 4).

After a calibration procedure described before, the ultrasonic time of flight was measured repeatedly in specific measurement points at the plane sides of the cores. The ultrasonic pulse velocity was then calculated based on the corrected time of flight measurement.

All laboratories performed ultrasonic measurements on 20 concrete cores. An additional homogeneous polyamide specimen was additionally measured following the same procedure. This polyamide specimen can be used as a reference specimen.

**Fig. 5:**
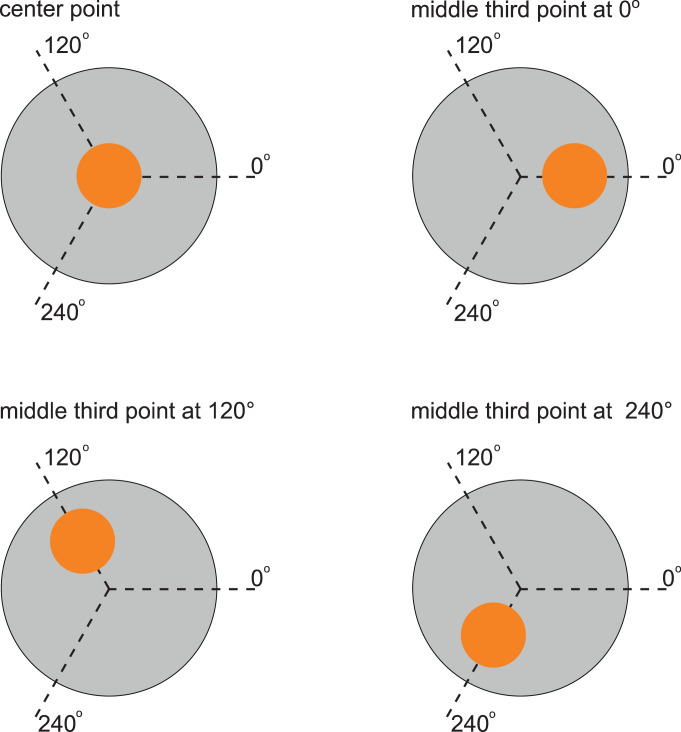
Measuring points.

A total of twelve ultrasonic measurements per specimen were performed. Six measurements were performed in transmission measurement in the center of the sample on the front surface. A total of 6 additional measurements were taken 2.5cm away from the center point (2 repetitions x 3 measurement points with azimuth = {0°, 120°, 240 °}). The measurement sequence for each specimen was performed in the following order (Fig. 5):

1. Measurement: Center point (CP)
2. Measurement: Middle third point (TP) at 0
3. Measurement: Center point (CP)
4. Measurement: Middle third point (TP) at 120
5. Measurement: Center point (CP)
6. Measurement: Middle third point (TP) at 240
7. Measurement: Center point (CP)
8. Measurement: Middle third point (TP) at 120
9. Measurement: Center point (CP)
10. Measurement: Middle third point (TP) at 240
11. Measurement: Center point (CP)
12. Measurement: Middle third point (TP) at 240

### 5.3 Rebound numbers on cubes

**Fig. 6:**
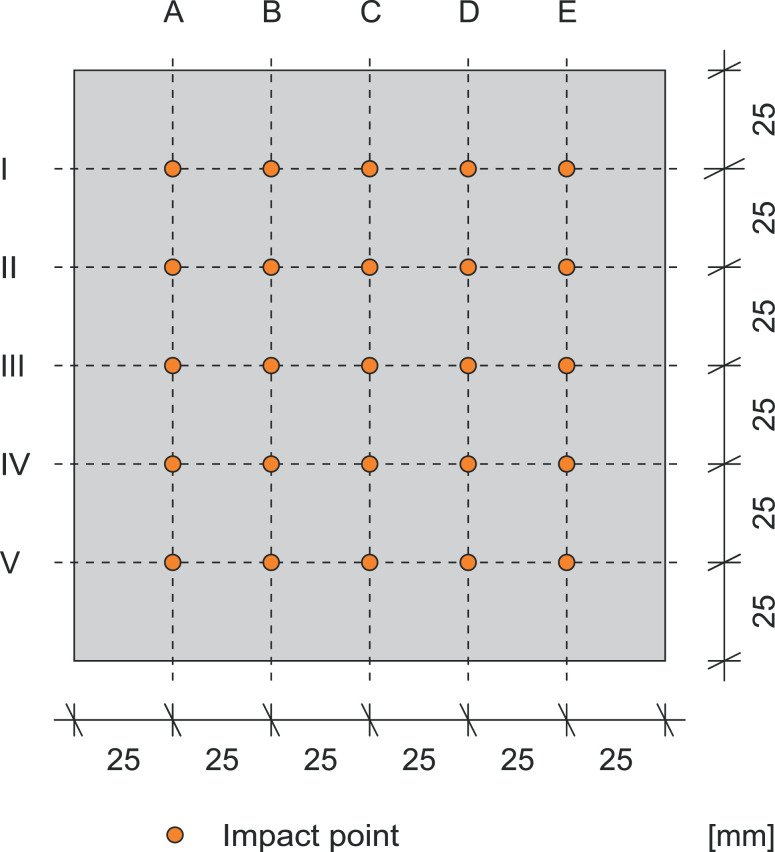
Side surface of a cube with impact pattern.

The areas of testing are two of the six surface areas of the cube. Each surface is divided into a pattern of 5×5 impact points. The columns are named from A to E, while the lines are numbered by Roman numerals from I to VI. The distance between the impact points and the edge is 25mm. Fig. 6 shows the impact pattern of one cube surface. Each point may only be hit once! Each laboratory tests five different cubes from the same mixture. The results are two datasets of 25 rebound numbers per cube. During the data acquisition, the cubes were fixed in a testing machine with 60kN to secure their position. All rebound hammers were checked for calibration on the test anvil before and after the test and passed den test.

### 5.4 Ultrasonic pulse velocity on cubes

**Fig. 7:**
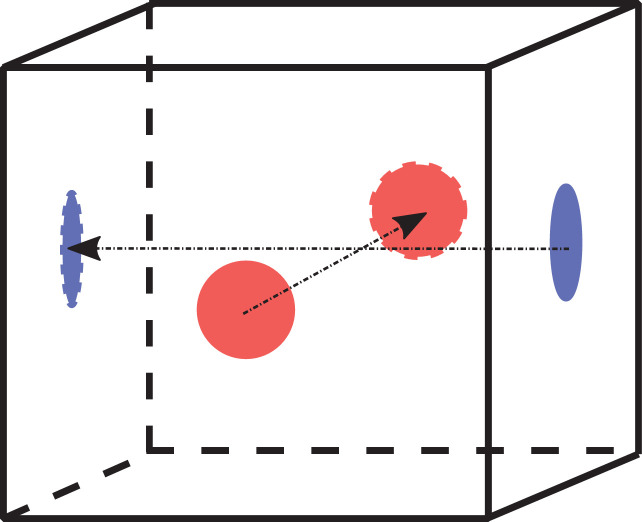
Measuring plan scheme on two opposite faces of a cube sample.

The measurement was performed longitudinally along the axis of the cores and along two opposite faces in the case of the cubes. One laboratory performed for each of the 25 cube samples, measurements at two opposite faces (Fig. 7) labeled as face with and without points according to the impact pattern of the rebound hammer tests.

### 5.5 Compressive strength

The compressive strength was determined in destructive tests according to DIN EN 12,504–1 [4] for the drilled cores and DIN EN 12,390–3 [5] for the cubes. The dimensions fulfill the requirements of DIN EN 12,504–1 [4] for the drilled cores and DIN EN 12,390–1 [7] for the cubes.

## Ethics Statements

These data do not include any human subjects, animal experiment, or social media platforms.

## CRediT Author Statement

**Daniel Gebauer:** Methodology, Investigation, Data Curation, Writing - Original Draft, Visualization, Project administration. **Raúl Enrique Beltrán Gutiérrez:** Methodology, Investigation, Data Curation, Writing - Original Draft, Visualization, Project administration. **Steffen Marx:** Conceptualization, Methodology, Resources, Supervision, Funding acquisition. **Marko Butler:** Methodology, Investigation, Writing - Review & Editing. **Konrad Grahl:** Investigation, Writing - Review & Editing. **Thomas Thiel:** Investigation, Writing - Review & Editing. **Stefan Maack:** Methodology, Investigation, Resources, Writing - Review & Editing. **Stefan Küttenbaum:** Investigation, Writing - Review & Editing. **Stephan Pirskawetz:** Investigation, Writing - Review & Editing. **Wolfgang Breit:** Methodology, Investigation, Writing - Review & Editing. **Martin Schickert:** Methodology, Investigation, Writing - Review & Editing. **Marco Krüger:** Investigation, Writing - Review & Editing.

## Declaration of Competing Interest

The authors declare that they have no known competing financial interests or personal relationships that could have appeared to influence the work reported in this paper.

## Data Availability

Interrelated Data Set from Nondestructive and Destructive Material Testing of Concrete Compressive Strength Specimens (Original data) (Dataverse).
